# Impact of Musashi-1 and Musashi-2 Double Knockdown on Notch Signaling and the Pathogenesis of Endometriosis

**DOI:** 10.3390/ijms23052851

**Published:** 2022-03-05

**Authors:** Theresa Strauß, Burkhard Greve, Michael Gabriel, Nurjannah Achmad, Dhanusha Schwan, Nancy Adriana Espinoza-Sanchez, Antonio Simone Laganà, Ludwig Kiesel, Matti Poutanen, Martin Götte, Sebastian Daniel Schäfer

**Affiliations:** 1 Department of Gynecology and Obstetrics, University Hospital Muenster, Albert-Schweitzer-Campus 1, Building A1, 48149 Münster, Germany; theresa.strauss@me.com (T.S.); nurjannah.achmad@outlook.com (N.A.); dhanusha.schwan@uk-essen.de (D.S.); nancyadriana.espinozasanchez@ukmuenster.de (N.A.E.-S.); ludwig.kiesel@ukmuenster.de (L.K.); sd.schaefer@ukmuenster.de (S.D.S.); 2 Department of Radiotherapy-Radiooncology, University Hospital Muenster, Albert-Schweitzer-Campus 1, Building A1, 48149 Muenster, Germany; greveb@uni-muenster.de; 3 Department of Obstetrics and Gyneacology, Institute of Medicine, University of Turku, 20014 Turku, Finland; micawo@utu.fi; 4 Research Centre for Integrative Physiology and Pharmacology, Institute of Biomedicine, University of Turku, 20520 Turku, Finland; matpou@utu.fi; 5 Department of Obstetrics and Gynecology, “Filippo Del Ponte” Hospital, University of Insubria, 21100 Varese, Italy; antoniosimone.lagana@uninsubria.it

**Keywords:** Musashi, Notch, stem cells, endometriosis, apoptosis, HES-1, KLF-4, Notch-2, SOX-2, ALDH

## Abstract

The stem cell marker and RNA-binding protein Musashi-1 is overexpressed in endometriosis. Musashi-1-siRNA knockdown in Ishikawa cells altered the expression of stem cell related genes, such as OCT-4. To investigate the role of both human Musashi homologues (MSI-1 and MSI-2) in the pathogenesis of endometriosis, immortalized endometriotic 12-Z cells and primary endometriotic stroma cells were treated with Musashi-1- and Musashi-2-siRNA. Subsequently, the impact on cell proliferation, cell apoptosis, cell necrosis, spheroid formation, stem cell phenotype and the Notch signaling pathway was studied in vitro. Using the ENDOMET Turku Endometriosis database, the gene expression of stem cell markers and Notch signaling pathway constituents were analyzed according to localization of the endometriosis lesions. The database analysis demonstrated that expression of Musashi and Notch pathway-related genes are dysregulated in patients with endometriosis. Musashi-1/2-double-knockdown increased apoptosis and necrosis and reduced stem cell gene expression, cell proliferation, and the formation of spheroids. Musashi silencing increased the expression of the anti-proliferation mediator p21. Our findings suggest the therapeutic potential of targeting the Musashi–Notch axis. We conclude that the Musashi genes have an impact on Notch signaling and the pathogenesis of endometriosis through the downregulation of proliferation, stemness characteristics and the upregulation of apoptosis, necrosis and of the cell cycle regulator p21.

## 1. Introduction

Endometriosis is defined by the presence of endometrium-like tissue outside the uterine cavity associated with stroma. Between 6% and 10% of women of reproductive age are affected. Endometriosis is an estrogen-dependent condition with chronic inflammatory characteristics [1]. Typical symptoms include dysmenorrhea, dyspareunia, as well as other pain symptoms linked to certain localizations of endometriotic lesions and subfertility [1,2]. 

Established treatment options include surgical removal of lesions and hormonal therapy. Both options do not guarantee the healing of the condition and can be associated with significant side effects, such as adhesion formation and hypoestrogenic symptoms [1,2,3]. The pathogenesis of endometriosis has been the subject of recent research. There seems to be a role for retrograde menstruation, coelomic metaplasia, lymphovascular metastasis and the embryonic rest theory in the development of endometriosis. The underlying molecular mechanisms still remain unclear and are complex, including the dysregulation of the endocrine milieu, chronic inflammation, aberrant angiogenesis and the dysregulation of factors associated with invasive growth [1]. Recent research points at an altered endometrial stem cell function as an additional pathogenetic route that is compatible with the classical concepts of endometriosis [4,5,6]. Notch signaling has been shown to be a relevant stemness-related pathway in endometriosis. It is more active in deep infiltrating endometriotic lesions of patients than in controls [5]. Glandular notch-1 expression is upregulated in the eutopic endometrium of patients suffering from deep infiltrating endometriosis compared with the endometrium of an endometriosis-free in vitro fertilization collective [7]. In contrast to this, another study found decreased notch signaling in the eutopic endometrium of women with endometriosis, resulting in impaired decidualization [8]. Notch activation was linked to progesterone resistance in endometriotic lesions [9] and to angiogenesis in a mouse model [10]. 

The Notch pathway is modulated by Musashi proteins, RNA binding proteins that act as translational repressors [11,12]. Musashi exists in two variants, Musashi-1 (MSI-1) and Musashi-2 (MSI-2). Both variants regulate the stem cell character of somatic and germ cells through effects on differentiation, survival, proliferation, and therapeutic resistance [13,14]. MSI-1 has been demonstrated to modulate development of endometrial cancer [15] and to be significantly upregulated in endometriotic tissue [6]. The dual knockdown of MSI-1 and MSI-2 in ovarian and breast cancer cells led to attenuation of stemness and therapy resistance [16,17]. As these data suggest a potential mechanistic involvement of Musashi proteins in endometriosis, the aim of this study is to elucidate the effect of MSI-1 and MSI-2 knockdown on endometriosis development in vitro. 

## 2. Results

### 2.1. Expression of Notch Signaling- and Stemness-Related Genes in Endometriosis

**Figure 1:**
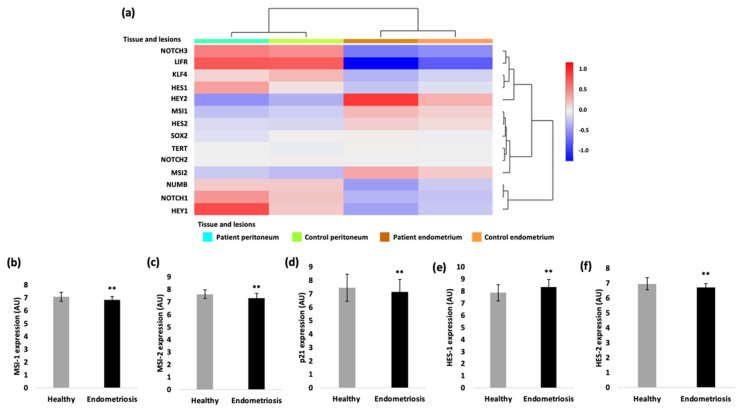
Gene expression analysis with the ENDOMET Turku Endometriosis Database. (**a**) The expression of the analyzed genes is depending on the tissue origin (peritoneum vs. endometrial tissue). The Musashi-1 expression (**b**), the Musashi-2 expression (**c**), the p21 expression (**d**) and the HES-2 expression (**f**) are in general decreased in endometriotic tissue compared to the healthy ones. The expression of the transcription factor HES-1 is increased in endometriotic cells compared to the healthy samples (**e**). For the gene expression of HES-2 and MSI-2 82, samples of healthy tissue and 576 samples of patients with endometriosis were analyzed. A total of 41 healthy samples were analyzed for the gene expression of HES-1 and HES-2 and 287 endometriotic samples for the MSI-1 and 288 samples for the HES-1 expression. (** *p* < 0.01, error bars = SD).

To analyze the gene expression of our genes of interest in endometriotic tissue, the ENDOMET Turku Endometriosis Database [18] was used. We performed database analyses in up to 576 endometriotic samples of ectopic lesions of endometriosis patients and up to 82 healthy control samples. The expression of different genes of the Notch signaling pathway (MSI-1, MSI-2, numb, Notch receptors, Notch-dependent transcription factors Hes, Hey and stemness markers LIFR, KLF-4, SOX-2) were first analyzed for their expression in different tissue types and different types of endometriotic lesions. Comparing the gene expression in the peritoneum and endometrial tissue of endometriosis patients and controls, the database analysis showed that the gene expression depends on the analyzed tissue, and that it is differentially altered between patients and controls (Figure 1a). Specifically, the expression of MSI-1, MSI-2, of the transcription factor HES-2 and the cell cycle regulator p21 are significantly reduced in patients with endometriosis according to the data (Figure 1). In contrast, the transcription factor HES-1 is overexpressed in patients with endometriosis compared to the healthy samples (Figure 1e). The dysregulated expression of the Musashi genes and Notch signaling constituents suggests that they may contribute to the pathogenesis of endometriosis. 

### 2.2. Loss of Musashi-1 and -2 Function Inhibits Cell Proliferation and Promotes Apoptosis and Necrosis in Endometriotic 12-Z Cells and Primary Endometriotic Stroma Cells 

**Figure 2:**
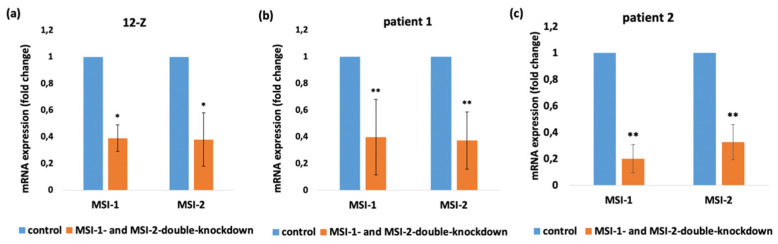
Confirmation of Musashi-knockdown in 12-Z cells and patient-derived endometriotic stroma cells. siRNA-mediated knockdown of MSI-1 and MSI-2 in 12-Z cells (**a**), patient 1 primary cells (**b**), and patient 2 primary cells (**c**) lead to the significant transcriptional downregulation of MSI-1 and MSI-2. * *p* < 0.05, ** *p* < 0.01, *n* > 3, error bars = SD.

As previously shown, the number of cells expressing the stem-cell-related gene MSI-1 is increased in endometriotic tissue compared to healthy endometrium [6]. According to the previous results, Musashi expressing progenitor cells could play an important role in the pathogenesis of endometriosis. To demonstrate the role of Musashi in endometriotic tissue at the functional level, we employed the siRNA technology to silence MSI-1 and MSI-2 in 12-Z cells and primary endometriotic stroma cells. Both homologs of Musashi were simultaneously downregulated to exclude potential compensatory effects [16,17]. Prior to analyses, the efficiency of the siRNA mediated double knockdown of MSI-1 and MSI-2 12-Z cells was controlled by a RT-PCR analysis, confirming the knockdown efficiency of 61% for MSI-1 and 62% of MSI-2 in 12-Z cells, for 60% for MSI-1 and 63% for MSI-2 in patient 1 primary cells and for 80% of MSI-1 and 67% of MSI-2 in patient 2 primary cells compared to control si-RNA transfected cells (Figure 2a–c). The results, thus, confirmed the successful siRNA double-knockdown of MSI-1 and MSI-2 expression in 12-Z cells and patients’ cells. 

**Figure 3:**
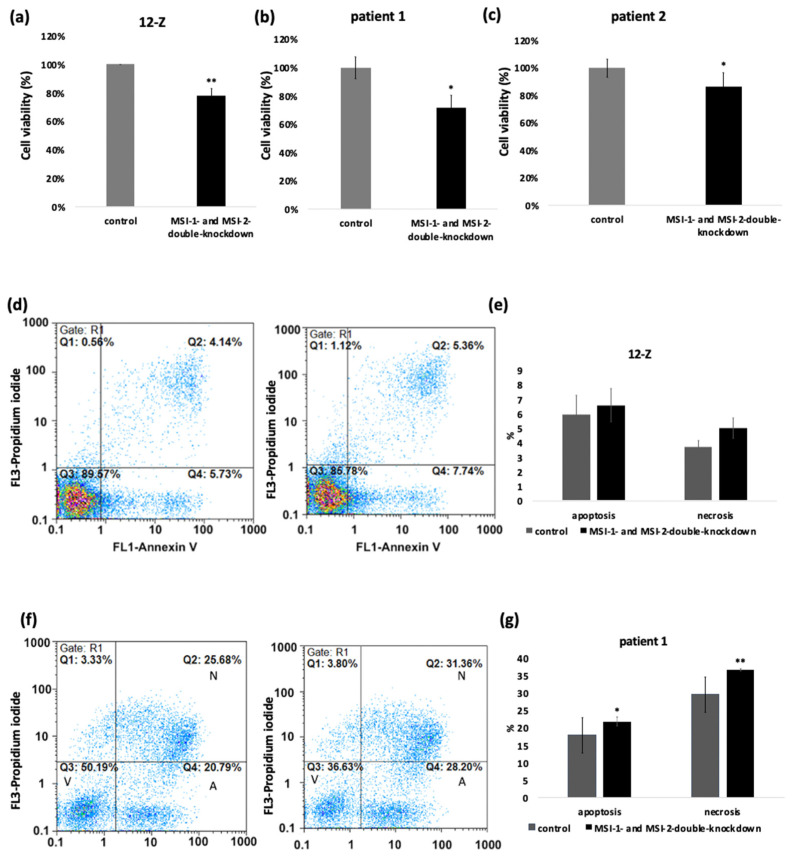
Musashi-double-knockdown effect on cell viability and apoptosis and necrosis rate. siRNA mediated knockdown of MSI-1 and MSI-2 in 12-Z cells (**a**), patient 1 cells (**b**), and patient 2 cells (**c**) lead to a significant downregulation of the cell viability (*n* > 3, * *p* < 0.05, ** *p* < 0.01). (**d**) Representative example of the impact of Musashi double knockdown caused an increased number of apoptotic cells by 2.01% and necrotic cells by 1.22% in 12-Z cells. (**f**) In the representative example, the apoptotic rate increased through the Musashi double knockdown from 20.79% to 28.20% and the necrosis rate from 25.68% to 31.36% in patient 1 cells. The 12-Z cells (**e**) showed an increased apoptotic and necrotic rate in each experiment, but according to viability no significance was detected. Patient 1 cells (**g**) showed a significant increase in the apoptotic and necrotic rate through Musashi double knockdown. (*n* = 3, * *p* < 0.05, ** *p* < 0.01, error bars = SD).

We next studied the impact of Musashi knockdown on several cellular phenotypes relevant to the pathogenesis of endometriosis. The influence of the Musashi double knockdown on metabolic activity as a readout of cell viability of 12-Z and primary cells was measured with MTT-Assay. The data showed that Musashi double knockdown had an influence on cell survival. The cells which were treated with MSI-1- and MSI-2-siRNA showed a reduced cell viability compared to the control-siRNA treated cells. The 12-Z cells showed a significant reduction in cell viability by 20%. Patient 1 cells showed a decreased cell viability by 28% and patient 2 cells on 14% (Figure 3a–c). Accordingly, the Musashi double knockdown in 12-Z cells and the cells of patient 1 resulted in an increased apoptosis and necrosis rate. The number of apoptotic cells was increased from 5.94% to 6.59% and the number of necrotic cells from 3.72% to 5.01 % in the 12-Z cells (Figure 3d,e). The proportion of apoptotic cells in patient 1 cells significantly increased from 18% to 22% and the proportion of necrotic cells from 29% to 37% (Figure 3f,g). The cells of patient 2 showed a similar increase apoptosis and necrosis rate in three of four experiments (results not shown). 

### 2.3. Loss of Musashi-1 and -2 Function Affects the Formation of 3D Spheroids 

**Figure 4:**
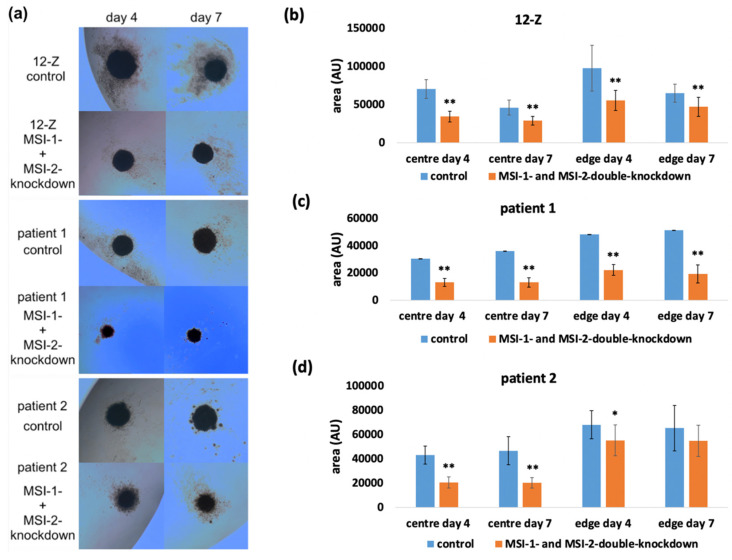
Impact of Musashi double knockdown on spheroid formation by endometriotic cells. (**a**) Images of spheroids of 12-Z, patient 1 and patient 2 cells on day 4 and day 7, which were formed by using the hanging drop method. (**b**) The 12-Z control spheroids were significantly larger compared to the Musashi-double-knockdown spheroids (*n* = 13, students *t*-test). (**c**) The patient 1 control spheroids were significantly larger on day 4 and day 7 in the center and edge of the spheroid compared to the Musashi-double-knockdown spheroids (*n* = 13, students *t*-test). (**d**) The patient 2 control spheroids showed a significantly larger size on day 4 and day 7 in the center compared to the Musashi-double-knockdown spheroids. The edge of the Musashi double knockdown was reduced (*n* = 13; Student’s *t*-test). For all figures in the panel * *p* < 0.05, ** *p* < 0.01, error bars = SD.

The impact of the Musashi double knockdown on the formation of spheroids as readout of stem-cell-related properties was analyzed using the hanging drop method. The spheroid size was measured and analyzed on day 4 and 7. The images in Figure 4a show that the 12-Z cells as well as the primary cells were capable of self-organizing into spheroids. Spheroids consisted of a solid core and were surrounded by a more diffuse margin of cells. The center and diffuse edge were analyzed separately in the knockdown experiments. The morphology of the spheroids was similar, but the 12-Z spheroids were larger than the primary stromal cell spheroids and the edge around the center of the 12-Z spheroids was more diffuse than in primary cells spheroids. To exclude cell counting errors, the size of the spheroids was measured in three different, independent experiments. Image J-based quantification revealed that the Musashi-double-knockdown cells showed a significantly reduced formation of the spheroids with a smaller size on day 4 and day 7 in the center and the diffuse edge around the center of the spheroid in 12-Z cells and patient 1 and patient 2 cells (Figure 4b–d).

### 2.4. Loss of Musashi-1 and -2 Function Inhibits ALDH-Activity and Reduces the Side Population

**Figure 5:**
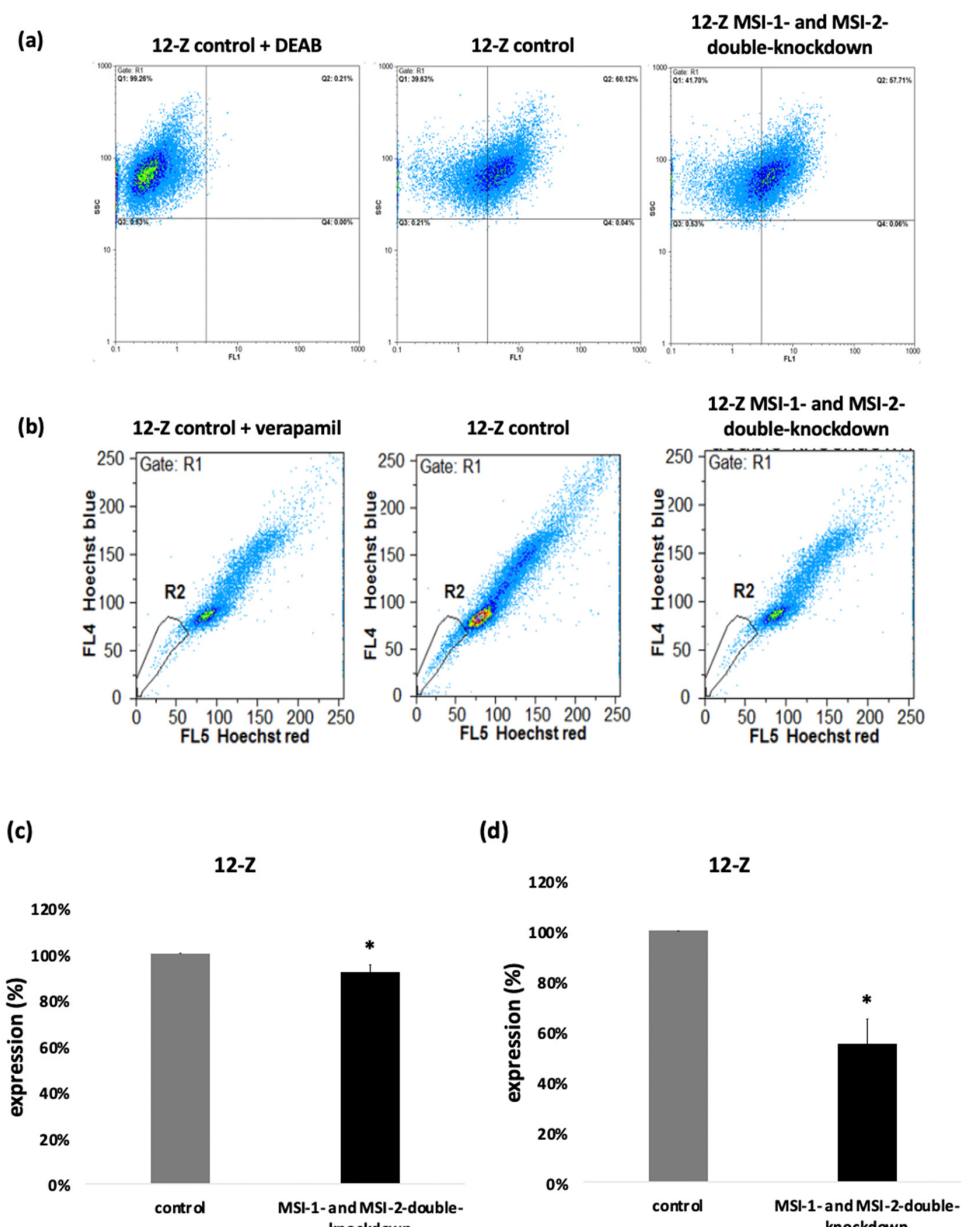
Musashi double knockdown affects stem cell characteristics. (**a**) The impact of the Musashi double knockdown on the ALDH activity of the 12-Z cells as a readout of stem cell activity is shown in the representative example in which the ALDH positive cell population was reduced by 8.66%. (**b**) Flow cytometry reveals a decreased side population (SP), a surrogate stem cell marker, after Musashi double knockdown. The side population cells are detected in gate R2. One sample was treated with verapamil, which inhibits ABC transporters. The side population of the verapamil treated cells in this representative example was 1.49% (left panel). The cells treated with the control si-RNA show 2.36% of cell in the side population (central panel). Musashi double knockdown reduced the side population to 0.6%. The downregulation of side population was 1.71%. (**c**) Quantitative analysis showed that Musashi double knockdown reduces the proportion of the ALDH positive cells to 91.8% (*n* = 3, * *p* < 0.05, error bars = SEM). (**d**) The impact of Musashi double knockdown on the SP phenotype was quantitatively analyzed and found to be reduced to 53.2% compared to controls (*n* = 3, * *p* < 0.05, error bars = SD).

To further analyze the impact of Musashi double knockdown on stem cell characteristics, the 12-Z cells were analyzed for ALDH activity and their SP phenotype as surrogate markers of a stem cell phenotype [4,19,20]. After incubation of the 12-Z cells with a fluorescent ALDH substrate in the presence or absence of the inhibitor DEAB followed by flow cytometry, Musashi double knockdown leads to a reduction in ALDH positive cells as presented in the representative example (Figure 5a). Through Musashi double knockdown, the pool of ALDH positive 12-Z cells was significantly reduced to 91.8% (Figure 5). Stem cells express a high level of ATP-binding cassette transporter proteins for example ABCG/Brcp, which are able to exclude the fluorescent dye Hoechst 33,342 from the cells, allowing for characterizing the side population phenotype via flow cytometry [19]. In a representative example, the 12-Z cells were detected to contain 2.36% SP cells and Musashi double knockdown reduced this population to 0.65% (Figure 5b). The ABC transporter inhibitor verapamil was used as a control to identify SP cells [19]. The quantitative analysis of three independent experiments revealed a significant reduction in the side population upon Musashi double knockdown by about 50% (Figure 5). 

### 2.5. siRNA Induced Double-Knockdown of Musashi-1 and -2 Leads to Altered Expression of HES-1, KLF-4, HES-2 and Notch-2 in Endometriotic Cells

**Figure 6:**
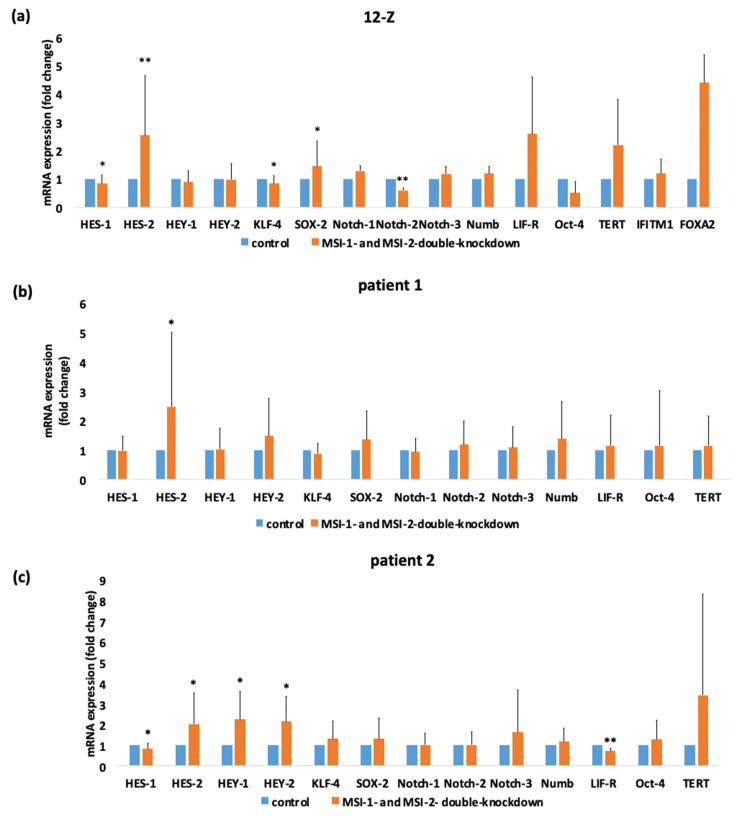
siRNA-mediated double knockdown of Musashi-1 and -2 expression results in significant alterations in stemness-related gene expression in 12-Z cells and primary endometriotic cells. 12-Z cells and primary cells were treated with MSI-1- and MSI-2-si-RNA or negative control si-RNA and 48 h after transfection, the mRNA expression of Notch signaling relevant genes and stem cell markers was analyzed via RT-PCR. (**a**) siRNA mediated the double knockdown of MSI-1 and MSI-2 results in a significant downregulation of KLF-4, HES-1, and Notch-2 and in a significant upregulation of HES-2 in 12-Z cells (*n* = 21). (**b**) In patient 1 cells, Musashi double knockdown leads to an upregulation of HES-1 expression (*n* = 15). (**c**) Downregulation of Musashi-1 and Musashi-2 in patient 2 cells represses the expression of the LIF-receptor and transcription factor HES-1 and increases expression of the transcription factors HES-2, HEY-1 and HEY-2 (*n* = 15). For all pictures in panel * *p* < 0.05, ** *p* < 0.01, error bars = SD.

To analyze a potential mechanism behind the altered phenotype of endometriotic cells due to Musashi double knockdown, the RT-qPCR of genes involved in Notch signaling (Notch-1, Notch-2, Notch-3, HES-1, HES-2, HEY-1, and HEY-2), in maintaining stem cell functions (KLF-4, OCT-4, and SOX-2) and in the pathogenesis of endometriosis (LIFR, Tert, IFITM1, and FOXA2) [1] was analyzed (Figure 6). Musashi double knockdown induced an upregulation of HES-2 in 12-Z and primary patient cells (2.54-fold in 12-Z, 2.48-fold in patient 1, 2.02-fold in patient 2). The mRNA expression of the transcription factors HES-1 and KLF-4 was significantly reduced through Musashi double knockdown in 12-Z cells (0.84-fold in 12-Z) and the mRNA expression of the Notch-2 receptor is highly significantly downregulated (0.84-fold 12-Z). In 12-Z cells, the expression of the transcription factor SOX-2 was significant upregulated (1.44-fold in 12-Z) (Figure 6a). The mRNA expression of HES-2 (2.48-fold in patient 1) was upregulated in patient 1 primary cells after treatment with MSI-1- and -2-si-RNA (Figure 6b). The downregulation of MSI-1 and MSI-2 in the primary cells of patient 2 cells repressed the expression of HES-1 (0.81 fold in patient 2) and of LIFR (0.7-fold in patient 2). The transcription factors HES-2 (2.02-fold in patient 2), HEY-1 (2.24 fold patient 2) and HEY-2 (2.14-fold patient 2) were upregulated through double-knockdown (Figure 6c). In 12-Z, patient 1 and patient 2 cells, no significant changes were detected for the factors Notch-1, Notch-3, Numb, OCT-4 and TERT. The transcription factor HES-2 was constantly upregulated in the investigated cells by Musashi double knockdown.

**Figure 7:**
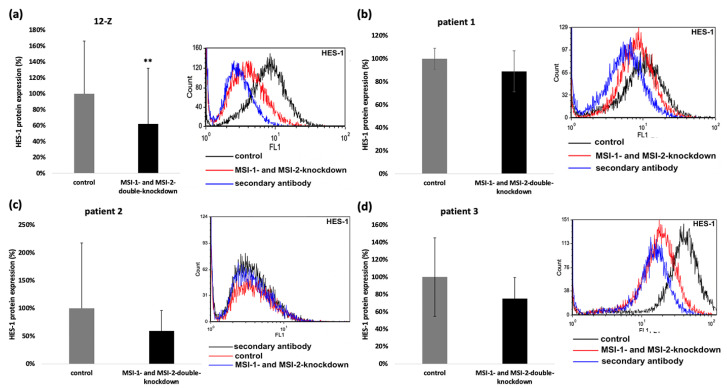
Musashi-depletion affects the expression of HES-1 protein. Musashi double knockdown effects HES-1-protein-expression. The 12-Z cells were transfected and 48 h later the flow cytometry analysis of HES-1 expression followed (**a**). The right panel shows a representative flow cytometry, and the left panel shows quantitative analysis of the 4 independent experiments that were performed (*n* = 4, ** *p* < 0.01). The impact of Musashi double knockdown on the HES-1 protein expression of patient 1 (**b**), patient 2 (**c**). and patient 3 (**d**) cells were also analyzed via flow cytometry. Downregulation of HES-1 was observed in all experiments of patient 1, 2 and 3, but due to high viability, no significant reduction was shown. On the left panel, the quantitative analysis is presented, and the right panel shows a representative flow cytometry (*n* = 3).

HES-1 is an important Notch-dependent transcription factor that has been linked to epithelial–mesenchymal transition, cell proliferation, migration, and invasion in endometriosis [21,22]. As HES-1 was downregulated on mRNA level, we investigated the HES-1 expression at the protein level via flow cytometry. HES-1 expression was downregulated in 12-Z cells after MSI-1- and MSI-2-si-RNA double knockdown compared to control siRNA treated cells (Figure 7a). In primary cells, the HES-1 expression was always individually downregulated, but the combined analysis did not lead to a significant resolution due to high viability (Figure 7b–d).

**Figure 8:**
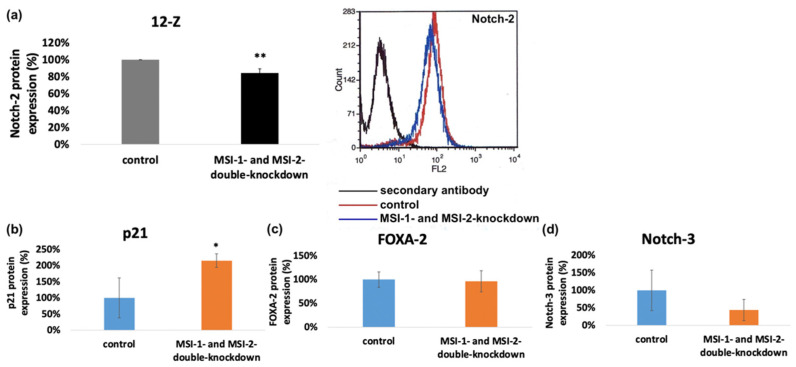
Impact of Musashi double knockdown on the protein expression of pathogenetic factors. (**a**) Flow cytometric analysis of protein expression of Notch-2 in endometriotic cells. Musashi double knockdown leads to a reduction in Notch-2 protein expression to a level of 84.5% in 12-Z cells (*n* = 3). (**b**) Quantitative analysis of Western blot results and the impact of Musashi double knockdown on the protein expression of p21 ((**b**), *n* = 5), FOXA-2 ((**c**), *n* = 4) and Notch-3 ((**d**), *n* = 3) (* *p* < 0.05, in 12Z cells. ** *p* < 0.01, error bars = SD).

Musashi double knockdown reduced the mRNA expression of Notch-2 in 12-Z cells (Figure 6a). According to the findings, the protein expression of Notch-2 in 12-Z cells was analyzed via flow cytometry and found to be significantly downregulated in 12-Z cells, following Musashi double knockdown (Figure 8a). 

To control the influence of Musashi double knockdown on the expression of further proteins possibly related to the observed phenotypic changes, Western blotting was performed with FOXA2-, Notch-3- and p21^WAF1/CIP1^-antibodies in the 12-Z and primary cells. Consistent with previous data on Ishikawa endometrial carcinoma cells subjected to MSI-1 siRNA knockdown [15], the cell cycle regulator p21^WAF1/CIP1^ was upregulated in MSI-1 and -2 depleted 12-Z cells compared to controls (Figure 8b and Supplement) [23]. In the case of Notch-3 and FOXA2, no consistent difference in protein expression was observed (Figure 8c,d and Supplementary Materials).

## 3. Discussion

In this in vitro study, we investigated the effect of a Musashi double knockdown on stem cell properties, apoptosis, necrosis, cell viability and gene expression of members of the Notch signaling pathway. The findings of this study demonstrate that the Musashi proteins, which are up regulated in endometriosis [6], act as a functional modulator of endometriotic processes. Other studies have shown that the Notch pathway partly controls the endocrine and antiangiogenic aspects and stem cell properties of endometriotic tissue [24]. As Musashi activates Notch signaling through the post-transcriptional regulation of Numb [11], it could be assumed that Notch signaling is downregulated through Musashi double knockdown via siRNA transfection. According to the results of the ENDOMET database, the expression depends on the localization of the endometriotic lesions. Based on the upregulation of the Musashi proteins in endometriotic cells, a Musashi double knockdown was performed using siRNA. The positive effects of Musashi double knockdown have already been shown in breast and ovarian cancer cells, in which the Musashi knockdown induces apoptosis and reduces cell proliferation [16,17]. The surviving fraction in a colony formation assay was also reduced in estrogen-receptor positive breast cancer cells through Musashi double knockdown [25]. 

The exact pathogenesis of endometriosis is still not completely known. The pathogenesis of endometriosis is complex [1]. In accordance with the stem cell theory, different stem cell markers are more highly expressed in endometriotic tissue than in normal endometrium [1,26,27]. While our data establish a novel contribution of the MSI–Notch signaling pathway to pathogenetic processes, such as cell proliferation and stemness, as a possible prerequisite for persistent growth of ectopic lesions, other factors, such as altered immune surveillance resulting in chronic inflammation, are also part of the complex pathogenesis [1]. Through a MSI-1 knockdown, pluripotency-associated transcription factors, such as OCT-4, were downregulated at the mRNA level in the 12-Z cell line. The 12-Z cells express the Notch-1 receptor, Notch-4 receptor [13] and several stem cell markers, such as OCT-4, KLF-4, MSI-1, and SOX-2 [4], and have shown a positive ALDH activity and a positive side population phenotype [4]. Therefore, this cell line appears well-suited for analyzing stemness-associated phenotypes in endometriosis. Previous studies have furthermore shown that cells derived from endometriotic tissue with a side population phenotype are able to reconstitute the tissue of patients with endometriosis in an in vivo model [20]. Through Musashi double knockdown, the side population and the ALDH activity were significantly reduced, which relates to a downregulation of stemness characteristics. According to the findings, the cell viability and the formation of spheroids are also reduced through Musashi double knockdown. The downregulated cell viability through Musashi double knockdown also assumes a reduction in stem cell properties. Stem cells normally show an unlimited potential of proliferation [1,19,28]. In breast and endometrial cancer cells, a Musashi-1 knockdown downregulated proliferation and stem cell gene expression and increased the expression of the anti-proliferation mediator p21 and apoptosis [15]. Similar effects were observed upon Musashi double knockdown in breast and ovarian cancer cells [16,17,25]. Consistently, the effects of the Musashi-1 knockdown on p21 were also shown through Musashi double knockdown in the 12-Z cells, as there was an upregulation of p21 protein expression. 

The transcription factor HES-1 is a part of the hairy and enhancer of split family, which are with the HEY-family part of the Notch target genes. HES-1 is overexpressed in patients with endometriosis [9]. In our study, Musashi double knockdown led to a downregulation of the expression of HES-1 on the mRNA and protein levels. 

The results assume a participation of the Notch pathway on the pathogenesis of endometriosis. Notch signaling is more active in patients suffering from deep infiltrating endometriosis [5,7]. Notch-2 expression was reduced in 12-Z cells on mRNA and protein level through Musashi double knockdown. It was shown that the expression of Notch-1 receptor and Numb are increased in endometriotic tissue, indicating a possible role of this pathway in disease pathogenesis [7]. 

A limitation of this study is that we only investigated the different aspects in an in vitro system. However, our investigations with the spheroids have benefits compared to the 2D cell culture [29]. Previous studies showed that 12-Z cells are able to self-organize into spheroids and show a larger formation, sometimes exhibiting slightly branching morphology compared to St-T1b and endometriotic stroma cells [30,31]. In our study, Musashi double knockdown led to a reduced size of the area of the center and the edge of the spheroids in 12-Z and primary cells, which agrees with the reduced proliferation and reduced stem cell phenotype. 

One limitation of this in vitro study is that the primary cells are derived from the stromal compartment and the 12-Z cells are from the epithelial compartment [30,31,32]. The different results of the gene expression on mRNA levels could be explained by the different origin of the epithelial 12-Z-cells and the stromal endometriotic primary cells. Another explanation could be the different localizations of the biopsies from the patient cells and the different stages of the disease. 

In the epithelial 12-Z cells, the transcription factor SOX-2 was significantly upregulated through Musashi double knockdown. Previous studies showed that, in endometriotic tissue, SOX-2-positive cells are more frequent than in healthy control endometrium [33]. Other stemness related genes, such as REX-1, Nanog and OCT-4, are also increased expressed in women with ovarian endometriosis compared to normal endometrium [34]. SOX-2, KLF-4, c-Myc and OCT-4 are called the Yamanaka factors, which are able to reprogram differentiated cells into a pluripotent state [35]. KLF-4 was decreased through Musashi double knockdown in 12-Z cells. KLF-4 can also act as a tumor suppressor depending on the localization. Its expression depends on p21 [36]. It was shown in epidermal stem cells (of zebrafish) that KLF-4 promotes the cell proliferation by repressing p53 expression and preventing cdkn1a/p21 induction [37]. It is also part of the regulation of pathogenetically relevant processes, such as proliferation, apoptosis [38] and cell differentiation [39]. In endometriotic cells, KLF-4 expression is epigenetically regulated by microRNA miR-200b, resulting in altered stemness and proliferation [40].

In patient 2 primary cells, the expression of the LIF-receptor was decreased through the knockdown. LIF, which is part of the interleukin-6-family of cytokines, binds to the LIF-receptor and is able to induce a signal regarding pluripotency in murine embryonic stem cells [41]. The LIF-R was reduced in 12-Z cells and patient-derived endometriotic stroma cells after the treatment with gamma secretase inhibitor (GSI), assuming that it may cause unwanted fertility-related side effects [24]. As Musashi double knockdown leads to similar results, the unwanted fertility-related side effects could also be assumed, potentially restricting Musashi inhibition to the perspective treatment of non-fertility associated symptoms in endometriosis patients, or to postmenopausal patient subgroups. Notably, GSI is a reagent inhibiting proteolytic activation of notch signaling [24].

As only a few members of the Notch signaling pathway were inhibited by the Musashi double knockdown, a multimodal way of silencing the Notch pathway could be considered for an even more therapeutic effect in future studies. For example, other therapeutic targets, such as fruit- and vegetable-derived natural compounds modulating the Notch pathway, were already considered [42].

In conclusion, the MSI-1 and MSI-2 double knockdown has an impact on cell survival, stem cell traits and the proliferation of endometriotic cells. The present study underlines the potential role of MSI-1 and MSI-2 as therapeutic targets in endometriosis. Overall, these data suggest that the Notch signaling pathway has an important role in the pathogenesis of endometriosis.

## 4. Materials and Methods

### 4.1. Materials

Media, fetal calf serum (FCS), and tissue culture supplies were obtained from Gibco BRL/Thermo Fisher (Waltham, MA, U.S.A.). Unless stated otherwise, all chemicals were from Sigma (Deisenhofen, Germany).

### 4.2. Cell Culture

The immortalized human endometriotic cell line 12-Z [32] was used as a suitable model system for studying an endometriotic stem cell phenotype [4,24]. The cell line was kindly provided by Prof. Anna Starzinski-Powitz (Goethe-University, Frankfurt, Germany). The 12-Z cells originate from a biopsy of a peritoneal endometriotic lesion of a 37-year-old patient. For immortalization, the SV40-T-Antigen served as an inhibitor of apoptosis. They were cytokeratin-positive, estrogen receptor alpha and beta and progesterone receptor positive and aromatase P450 positive [32]. As the 12-Z cells are characterized by adherent growth and a confluent monolayer, the cells had to be split two times a week. The primary endometriotic stroma cells were obtained from three different patients with endometriosis undergoing laparoscopy at Münster University Hospital between October 2012 and March 2014. The patient 1 cells (OP-5) were obtained from an endometriotic lesion in the rectovaginal septum with a rASRM II score of a 35-year-old patient with multiple lesions in different locations. The patient 2 primary cells (OP-10) originated from a peritoneal superficial endometriotic lesion of the pelvic sidewall with an rASRM Score III of a 19-year-old patient. The patient 3 primary cells (OP-7) are from an endometriotic lesion of the uterine serosa of a 39-year-old woman. The endometriotic deposit was classified as rASRM II. The study was carried out in accordance with the Declaration of Helsinki and approved by the local ethics commission (Ethikkommission der Ärztekammer Westfalen-Lippe und der Medizinischen Fakultät der WWU; approval no. 1 IX Greb 1 from 19 September 2001, updated 2012). All participants gave written informed consent. No renumeration was offered to the patients to enter or continue the study. The isolation of the primary cells was performed as previously described [42]. The 12-Z cells and the primary endometriotic stroma cells were cultured in high glucose DMEM with 10% FCS and 1% penicillin/streptomycin. The cells were cultured in a humidified atmosphere of 5% CO_2_ at 37 °C. 

### 4.3. siRNA Transfection

One day before siRNA transfection, the cells were plated in six-well plates and incubated for 24 h at 37 °C to reach a confluency of 70–80%. The cells were either transfected with 10 nM each of MSI-1 (S8980) and MSI-2 siRNA (S42757) or negative control siRNA #1 (ThermoFisher Scientific, Waltham, MA, U.S.A.) in OPTI-MEM media (Life Technologies, Grand Island, NE, U.S.A.) via lipotransfection with Dharmafect^®^ reagent (Thermo Fisher Scientific, Waltham, MA, U.S.A.). After 24 h of incubation, OPTI-MEM media were replaced by a DMEM culture medium. Then, 24 h after media change, the cells were used for further experiments.

### 4.4. RNA Isolation and Reverse Transcription for cDNA Synthesis 

Forty-eight hours after transfection, mRNA isolation was performed according to the supplier’s protocol, using the InnuPREP^®^ RNA mini kit (Analytik Jena AG, Jena, Germany). cDNA synthesis was carried out according to the supplier’s protocol, using the High-Capacity cDNA Reverse Transcription Kit (Applied Biosystems, Foster City, CA, U.S.A.). Reverse transcription was performed on a TGradient thermocycler (Biometra, Göttingen, Germany).

### 4.5. Quantitative TaqMan Real-Time PCR

To control the Musashi knockdown efficiency, quantitative RT-PCR was performed using the ROX Probe Master Mix dTTP (Takyon™, cat. No. UF-RPMT-C0701, Eurogenetec, Seraing, Belgium). Per well, 20 ng cDNA were used. The RT-PCR was performed with the Peqlab Peqstar 96Q (VWR, Darmstadt, Germany) with 1 cycle at 50 °C for 2 min and then 40 cycles at 95 °C for 15 s and at 60 °C for 1 min. For analysis of the gene expression, actin was used as a housekeeping gene and the data of the target gene expression were normalized to the actin gene. The fold change was calculated using the Ct-values and 2-ΔΔCt-method. The used pre-designed TaqMan gene expression systems are listed in the Supplementary Table S1.

### 4.6. Quantitative SYBR Green RT-PCR

For gene expression analysis, quantitative RT-PCR was performed using ROX SYBR^®^ Master Mix blue dTTP (Takyon™, cat. No. UF-RSMT-B0701, Eurogenetec, Seraing, Belgium) and the primers listed in Table S2 in the Supplement. For the RT-PCR, 25 ng cDNA were used per well. For the RT-PCR, the Peqlab Peqstar 96Q (VWR, Darmstadt, Germany) was used. As a housekeeping gene, actin was used, and the fold change was determined using the 2-ΔΔCt-method and the Ct value. As a program for the RT-PCR, four different stages were used for one cycle. The first cycle was performed at 50 °C for 2 min, following 40 cycles of 15 s at 95 °C and of 1 min at 60 °C.

### 4.7. Western Blotting

Protein lysates for Western blotting were extracted from cultured cells 48 h after the siRNA transfection by using RIPA buffer as previously described [43]. A total of 20–30 µg of protein per lane were separated and loaded on a 10% tris-polyacrylamide gel and electrophoresed at 0.02 A for 15 min and 0.04 A for 60 min. The proteins were transferred to a Hybond nitrocellulose membrane (GE Healthcare, Munich, Germany) at 16 V for 50 min. The protein lanes and molecular weights were estimated by using Ponceau-S solution (Sigma-Aldrich^®^ Life Science, Taufkirchen, Germany). The membrane was blocked for 1 h with 2.5% non-fat dry milk and afterwards incubated for 16 h at 4 °C with one of the primary antibodies in Table S3 in the Supplement diluted in 10 mL 5% BSA. The membrane was washed 3x for 5 min with Tris-buffered saline and then incubated for 1 h with the depending secondary antibody (listed in Table S3 in the Supplement). After washing, the membrane 3x for 5 min with Tris-buffered saline, the membrane was incubated with an ECL reaction mix (SuperSignal^®^ West Pico Chemiluminescent Substrate by Thermo Scientific, Rockford, IL, U.S.A.). The signal was detected by the FlowMax. The membranes were stripped with 0.2 M NaCl (pH = 13.3), washed with H_2_O, and blocked for 1 h with 2.5% non-fat dry milk, before being re-incubated with the next primary antibody.

### 4.8. Cell Viability/Proliferation Assay 

Metabolic activity was assessed by MTT assay. The cells were seeded in 96-well plates 48 h after transfection. Then, 144 h after transfection, the cells were treated with methylthiazolyldiphenyltetrazolium bromide (MTT) as previously described [4]. The measurements were performed 24 h after the treatment at 495 nm on a VersaMax™ microplate reader (Molecular Devices, San Jose, CA, U.S.A.). Data are shown as a percentage of the absorption of the MSI-1 and MSI-2-treated cells over the control.

### 4.9. Flow Cytometric Measurement of HES-1 and Notch-2 Expression

The cells were removed from the well plates 48 h after transfection with 2 mmol/L EDTA in Ca^2+^/Mg^2+^-free PBS buffer and afterwards centrifuged for 5 min and 1200 rpm. In the case of Notch-2, 20 µL of a directly labelled antibody was used (BD Pharmingen, San Jose, CA, U.S.A.) to stain 1 × 10^6^ cells suspended in 100 µL PBS supplemented with 5% BSA. Cells were stained for 20 min in the dark, followed by addition of 900 µL PBS/BSA. Afterwards, flow cytometric measurement was performed. To quantify HES-1 expression, 1 × 10^6^ cells were washed with BD Perm/Wash Buffer (BD Pharmingen, San Jose, CA, U.S.A.) and centrifuged for 5 min at 1200 rpm. Afterwards the cells were incubated at 4 °C for 20 min with 250 µL BD Cytofix/Cytoperm™ (BD Pharmingen, San Jose, CA, U.S.A.). After washing the cells with BD Perm/Wash Buffer and centrifugation for 5 min and 1200 rpm, the samples were incubated with 20 µL of the primary antibody HES-1 (Santa Cruz Biotechnology, Santa Cruz, CA, U.S.A.) for 1 h at room temperature in the dark. A total of 20 µL PE isotype control antibodies or 20 µL BD Perm/Wash Buffer were added to the staining control samples. The caspase samples and their controls were ready to be measured: after the addition of 900 µL Perm/Wash Buffer, fluorescence intensity was detected in FL2. In the case of HES-1, two further washing steps were performed followed by addition of the secondary anti-mouse antibody Alexa-Fluor 488 (Invitrogen, Carlsbad, CA, U.S.A.) to all samples for one hour in the dark. After another two washing steps, the cells were suspended with 900 µL BD Perm/Wash and measured on a flow cytometer (CyFlow Space, Sysmex Partec, Münster, Germany). Excitation took place with a 488 nm argon laser and the emission of the fluorescence was measured at 527 nm in FL1 (Alexa 488).

### 4.10. Aldehyde Dehydrogenase Activity Assay

Furthermore, 24 h after the siRNA transfection with MSI-1 and MSI-2, the activity of the Aldehyde dehydrogenase (ALDH) was detected by using the ALDEFLUOR assay kit (STEMCELL Technology, Vancouver, BC, Canada), as previously described [15]. The ALDH activity was measured using a Cyflow Space cytometer (Sysmec/Partec, Görlitz, Germany) with a 488 nm argon laser for excitation. The emission of the fluorescence was measured at 527 nm in FL1. For the quantification of the fluorescence intensity, a quadrant gate was set in the dotplot of FL1 (*x*-axis) against side scatter SSC (*y*-axis) and the ALDH positive cells could be quantified in Q2 using the FloMax software (Quantum Analysis, Münster, Germany). The results were shown by the percentage of the mean of the ALDH-positive cells over the whole cell population. Per sample, 20,000 events were analyzed.

### 4.11. Side Population Analysis

A side population analysis was performed as previously described [15]. The measurement of side population phenotype is based on the upregulated expression of ABC transporter protein family members in stem cells. The phenotype corresponds to a surrogate marker of stemness. So, 72 h after siRNA transfection, the side population analysis was performed, using the Hoechst 33,342 dye exclusion technique. A total of 1 × 10^6^ cells were stained with 5 µg/mL Hoechst 33,342 (Sigma-Aldrich, Saint Louis, MO, U.S.A.) in DMEM containing 2% FCS at 37 °C for 90 min. Afterwards, 2 µg/mL propidium iodide was added to the cells for the detection of death cells. After the staining, the fluorescence emissions of the cells were analyzed on a flow cytometer (CyFlow Space, Sysmex Partec, Münster, Germany) using a 16 mW 375 nm UV laser. The measurement was made with the FloMax-software for data acquisition (Quantum Analysis, Münster, Germany). The fluorescence emission was shown as a dot-plot histogram with Hoechst blue on the *y*-axis and Hoechst red on the *x*-axis; the SP-cells reside in gate R2. The Hoechst signals were slivered using a 610 nm dichronic mirror into FL5 at 665 nm and FL4 at 455 nm. 

### 4.12. Annexin V Apoptosis Assay

For the detection of the apoptosis and necrosis rate, the cells were treated 48 h after siRNA transfection with the FITC Annexin V Apoptosis Detection Kit 1 (BD Pharmingen™, BD Biosciences, San Diego, CA, U.S.A.). Apoptosis was determined as detailed by the manufacturer’s manual. The measurements were performed on a flow cytometer (CyFlow Space, Sysmex Partec, Münster, Germany) with a 488 nm argon laser. Signals were collected at 665 nm in FL3 and at 527 nm in FL1. For visualizing and managing the flow data, the FloMax software (Quantum Analysis, Münster, Germany) was used. A total of 20,000 cells were analyzed per sample. The apoptotic cells were determined in the fourth quartile (Q4). Necrotic/late apoptotic cells were annexin V and propidium iodide positive because of their permeable membrane and presented in quadrants Q1 and Q2. The quadrant Q3 represents the viable cells.

### 4.13. Hanging Drop Assay

The hanging drop method was used for the generation of spheroids. A total of 20 µL drops containing 20,000 cells were deposited on the upper lid of a plastic Petri dish. The bottom of the Petri dish was filled with 10 mL PBS. The drops were incubated at 37 °C and 7.5% CO_2_ for 7 days. On day 4 and day 7, a photograph of the spheroids was taken using an Axiovert100 microscope (Carl Zeiss, Jena, Germany) with a 10x objective and an AxioCam MRc (Carl Zeiss, Jena, Germany) with the program AxioVision (Carl Zeiss, Jena, Germany). The experiment was repeated 3 times and per experiment 5 drops were analyzed. The area of the spheroids was analyzed using ImageJ. The center of the drop and the halo around the center of the spheroids were measured.

### 4.14. Statistical Analysis

The experiments were repeated independently at least 3 times with 2–3 biological replicates per run. The program Microsoft Excel was used for statistical analysis. Data were expressed as mean values +/−SD. The significance was calculated using the Student’s unpaired *t*-test for two samples with unequal variances (heteroskedastic). When reaching *p* < 0.05, the results were statistically significant and when *p* < 0.01 applied, the results were considered as highly significant.

## Data Availability

Patient gene expression datasets were retrieved and analyzed using the publicly accessible ENDOMET Turku Endometriosis Database (https://endometdb.utu.fi/, accessed on 15 December 2021) [18]. All experimental data presented in this study are contained in this manuscript.

## Supplementary Materials

The following supporting information can be downloaded at: https://www.mdpi.com/article/10.3390/ijms23052851/s1.
