# Supplementary material for: Impact of Musashi-1 and Musashi-2 Double Knockdown on Notch Signaling and the Pathogenesis of Endometriosis
Source: Int J Mol Sci. 2022 Mar 5;23(5):2851. doi: 10.3390/ijms23052851 (PMC8911246; doi:10.3390/ijms23052851)
Supplement: Supplementary file 1 [file ijms-23-02851-s001.zip › ijms-1600508-supplementary.pdf]

Supplementary file for

# Impact of Musashi-1 and Musashi-2 double knockdown on Notch signaling and the pathogenesis of endometriosis

**Theresa Strauß [1], Burkhard Greve [2], Michael Gabriel [3], Nurjannah Achmad [1§], Dhanusha Schwan [1#], Nancy Adriana Espinoza-Sanchez [1,2], Antonio Simone Laganà [4], Ludwig Kiesel [1], Matti Poutanen [5], Martin Götte [1*] and Sebastian Daniel Schäfer [1]**

1 Department of Gynecology and Obstetrics, University Hospital Muenster, Albert-Schweitzer-Campus 1, Building A1, 48149 Münster, Germany; tete.strau@gmail.com; nurjannah.achmad@outlook.com; Dhanusha.Schwan@uk-essen.de; Ludwig.kiesel@ukmuenster.de; nancyadriana.espinozasanchez@ukmuenster.de; sd.schaefer@ukmuenster.de
2 Department of Radiotherapy-Radiooncology, University Hospital Muenster, Albert-Schweitzer-Campus 1, Building A1, 48149 Muenster, Germany; greveb@uni-muenster.de; nancyadriana.espinozasanchez@ukmuenster.de
3 Department of Obstetrics and Gyneacology, Institute of Medicine and Research Centre for Integrative Physiology and Pharmacology, Institute of Biomedicine, University of Turku, Finland; micawo@utu.fi
4 Department of Obstetrics and Gynecology, "Filippo Del Ponte" Hospital, University of Insubria, Varese, Italy; antoniosimone.lagana@uninsubria.it
5 Research Centre for Integrative Physiology and Pharmacology, Institute of Biomedicine, University of Turku, Finland; matpou@utu.fi
§ present address: Jenderal Achmad Yani University, Cimahi, West Java, 40513 Indonesia
# present address: Klinische Forschergruppe 117, Universitätsklinikum Essen, Essen, Germany
* Correspondence: mgotte@uni-muenster.de

***Supplementary Table S1.*** *TaqMan assay IDs (Thermo Fisher)*

| **TaqMan-Sonden** | **Assay ID** |
|---|---|
| Aktin | Hs99999903 |
| MSI-1 | Hs00159291 |
| MSI-2 | Hs00292670 |

***Supplementary Table S2.*** *SYBR green PCR primer sequences*

| **SYBR green PCR primers** | **Primer sequence** |
|---|---|
| SOX-2 (forward primer)<br>SOX-2 (reverse primer) | TGG CGA ACC ATC TCT GTG GT<br>CCA ACG GTG TCA ACC TGC AT |
| AKTIN (forward primer)<br>AKTIN (reverse primer) | CAA AGA CCT GTA CGC CAA CAC<br>CAT ACT CCT GCT TGC TGA TCC |
| Notch-1 (forward primer)<br>Notch-1 (reverse primer) | GGT GAG ACC TGC CTGA ATG<br>GTT GGG GTC CTG GCA TC |
| Notch-2 (forward primer) | AAC TGT CAG ACC CTG GTG AAC |

| | |
|---|---|
| Notch-2 (reverse primer) | CGA CAA GTG TAG CCT CCA ATC |
| Notch-3 (forward primer)<br>Notch-3 (reverse primer) | TGT GCA AAT GGA GGT CGT<br>CCT GAG TGA CAG GGG TCC T |
| Numb (forward primer)<br>Numb (reverse primer) | GGCACCACAATCTCCTACCTTC<br>TGGTTTCACGCACAGGCATTGC |
| HES-1 (forward primer)<br>HES-1 (reverse primer) | GTGAAGCACCTCCGGAAC<br>CGTTCATGCACTCGCTGA |
| HES 2 (forward primer)<br>HES 2 (reverse primer) | AACCAGAGCCTGAGCCAGCTTA<br>TGCAGGAAGCGCACGGTCATTT |
| HEY-1 (forward primer)<br>HEY-1 (reverse primer) | GAGATCCTGCAGATGACCGT<br>AGATAACGCGCAACTTCTGC |
| HEY-2 (forward primer)<br>HEY-2 (reverse primer) | TTGCCCATGCCTAAACTAGTGC<br>TCTCACGTGCTTGATTTCAGCATA |
| KLF-4 (forward primer)<br>KLF-4 (reverse primer) | GCCTAAATGATGGTGCTTGGTG<br>GCAGTTTGAAAACTTTGGCTTCC |
| OCT-4 (forward primer)<br>OCT-4 (reverse primer | CTGGAGTTTGTGCCAGGGTTT<br>CTTCACCTTCCCTCCAACCAG |
| LIF-R (forward primer)<br>LIF-R (reverse primer) | TCTTGCGAGCCTATACAGAT<br>TCTGGATTTGGAATATCAGG |
| Tert (forward primer)<br>Tert (reverse primer) | GGAGCAAGTTGCAAAGCATTG<br>TCCCACGACGTAGTCCATGTT |

***Supplementary Table S3*** *Antibodies used for western blotting*

| **Primary antibody** (manufacturer) | **Secondary antibody** | **Dilution (1.Ab/2.Ab)** |
|---|---|---|
| Musashi-1 (Santa Cruz) | Anti-Mouse IgG | 1:200 |
| Musashi-2 (Abnova) | Anti-Mouse IgG | 1:2000 |
| p21 (Cell Signaling) | Anti-Rabbit IgG | 1:1.000 |
| FOXA2 (Cell Signaling) | Anti-Rabbit IgG | 1:1000 |
| Notch 3 (Cell Signaling) | Anti-Rabbit IgG | 1:1000 |
| Alpha-Tubulin (Santa Cruz) | Anti-Mouse IgG | 1:4.000 |

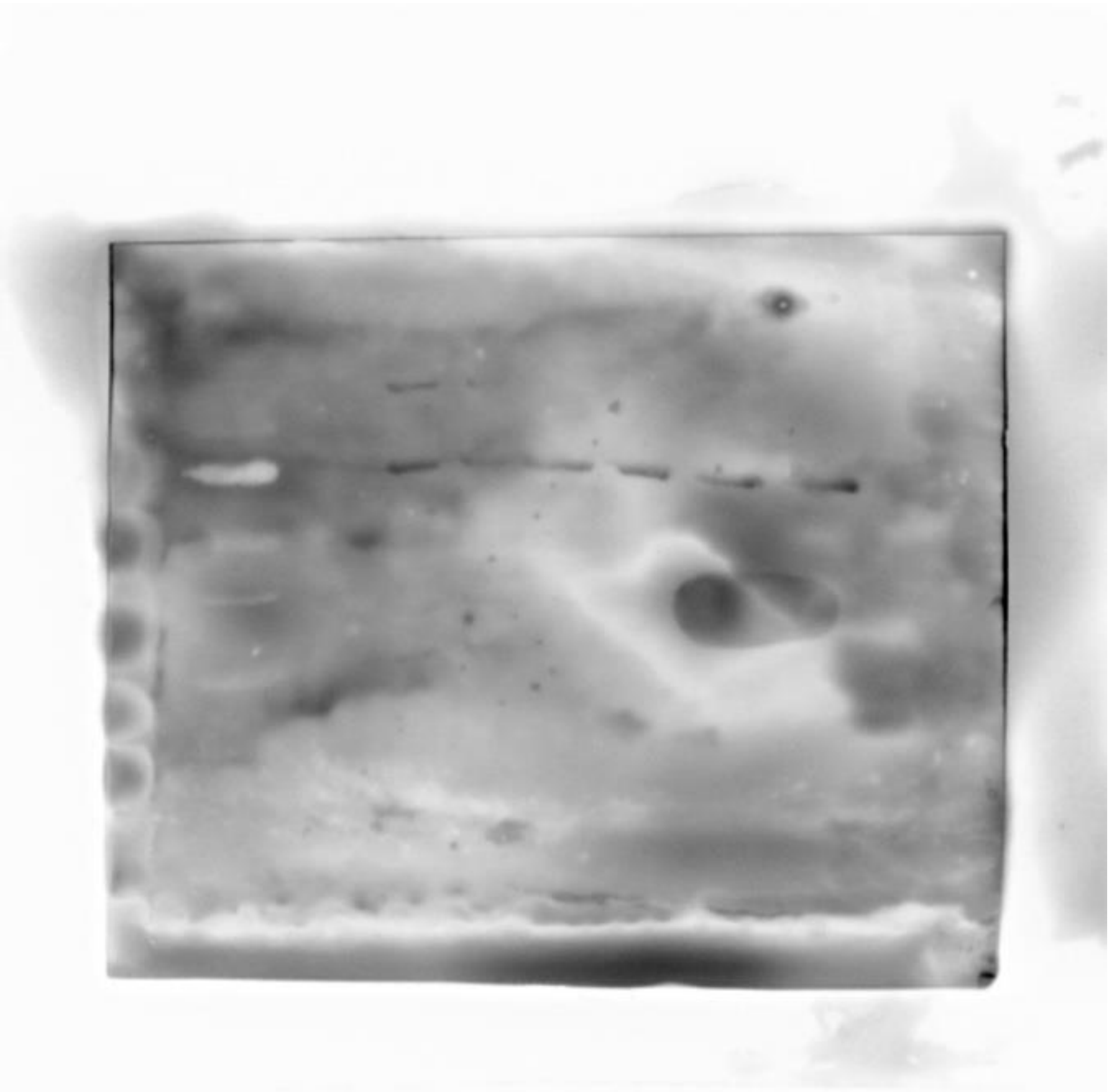

***Supplementary Figure S1**- Western Blot for Notch 3 - Lane 1: control 12-Z, lane 2: MSI-1- and MSI-2-double-knockdown, lane 3 and 4: patient 2 control, lane 5 and 6: patient 2 MSI-1- and MSI-2-double-knockdown, Left lane = Molecular weight markers*

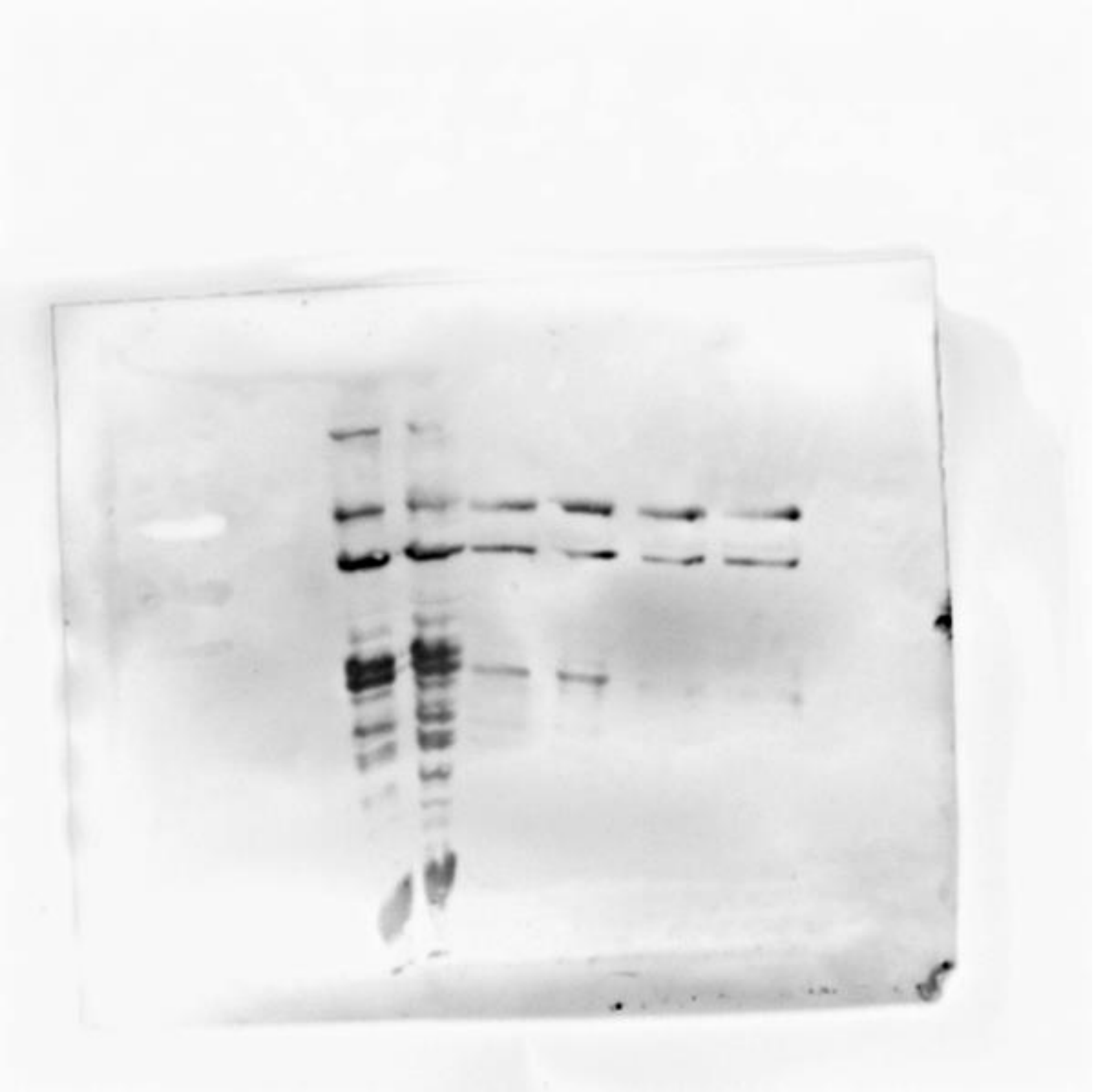

***Supplementary Figure S2**- Western Blot for Tubulin corresponding to samples shown in Fig. S1 - Lane 1: control 12-Z, lane 2: MSI-1- and MSI-2-double-knockdown, lane 3 and 4: patient 2 control, lane 5 and 6: patient 2 MSI-1- and MSI-2-double-knockdown Left lane = Molecular weight markers*

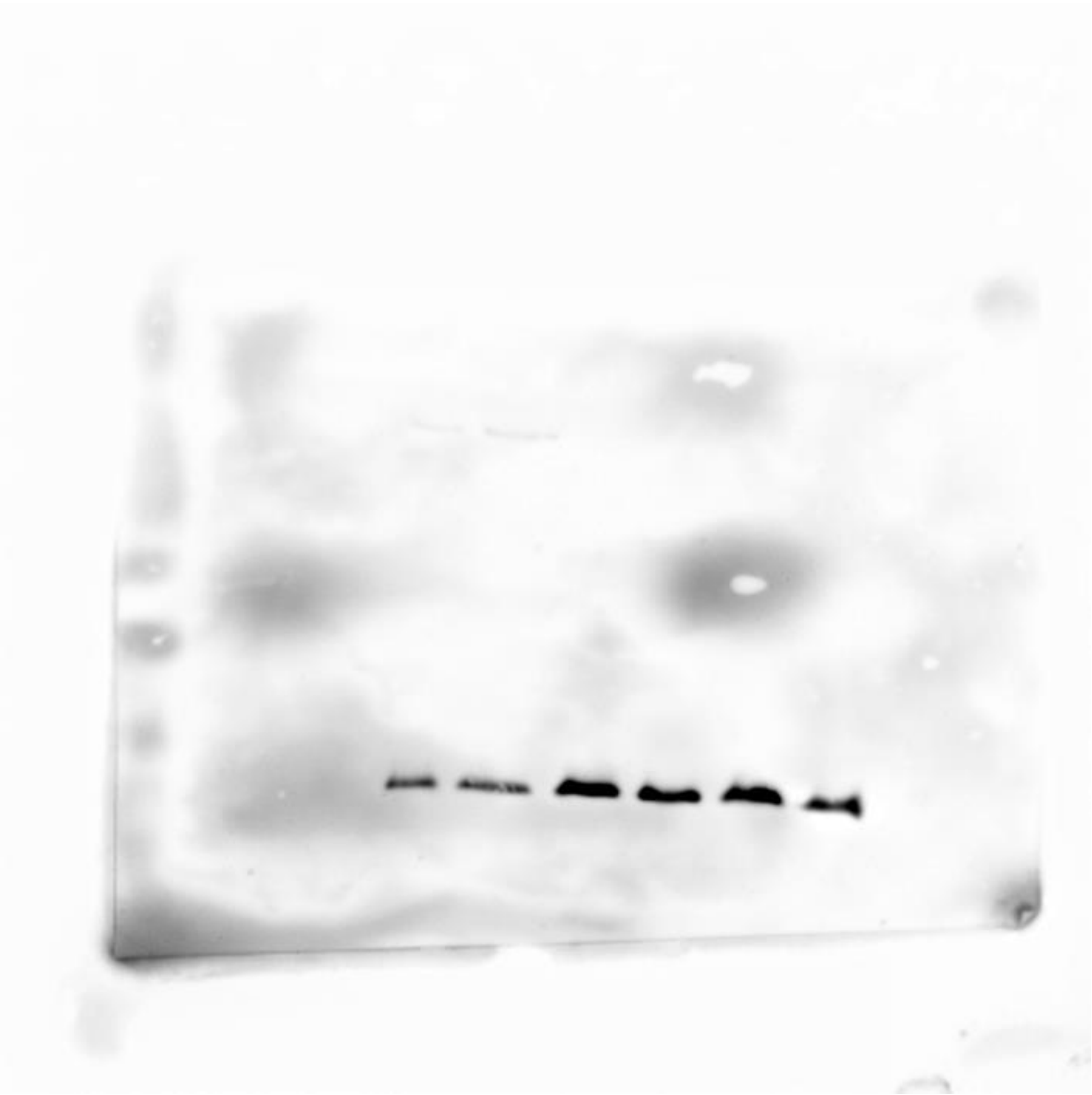

***Supplementary Figure S3-*** *Western Blot for Foxa2 - Lane 1: control 12-Z, lane 2: MSI-1- and MSI-2-double-knockdown, lane 3 and 4: patient 2 control, lane 5 and 6: patient 2 MSI-1- and MSI-2-double-knockdown*

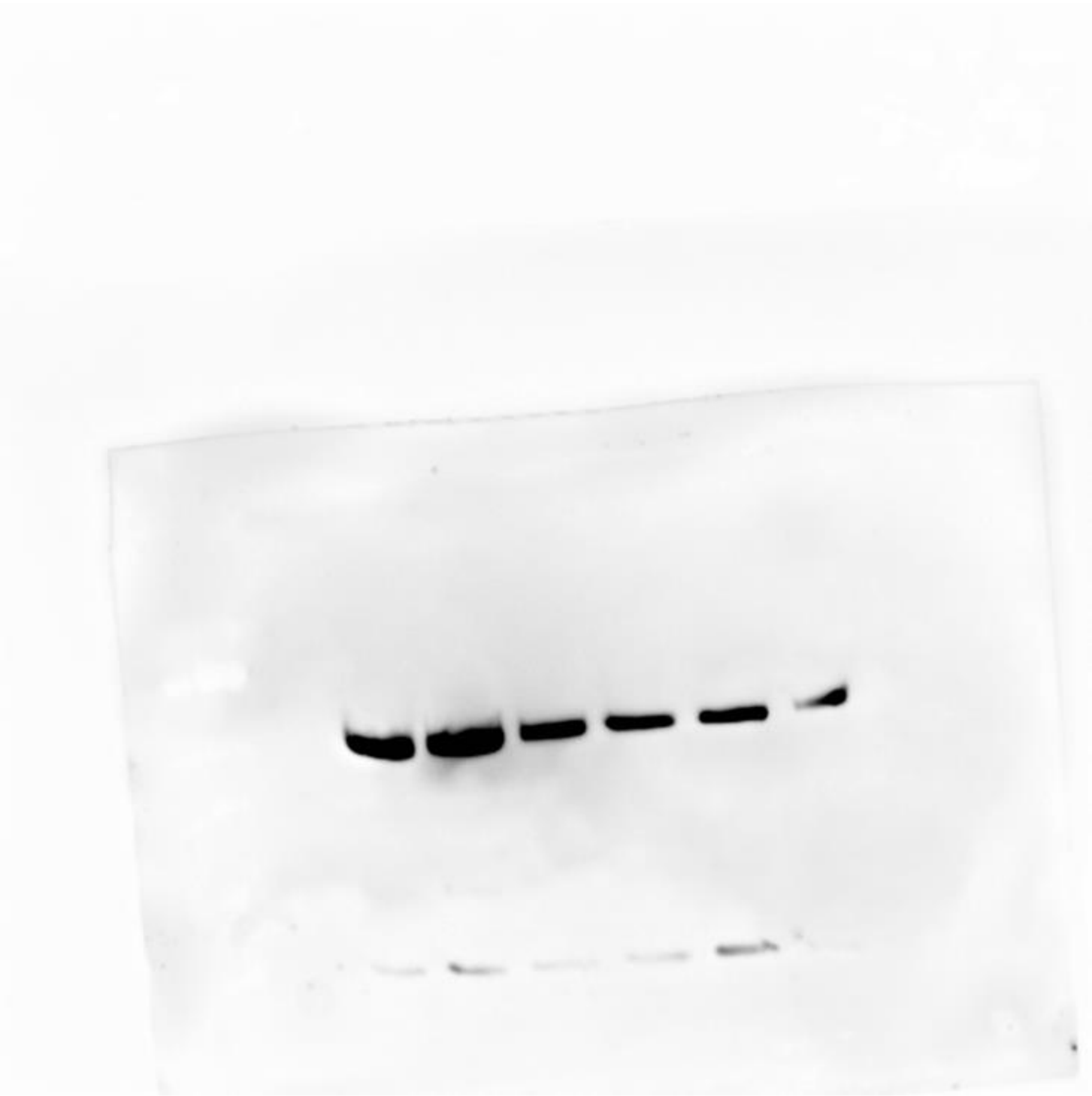

***Supplementary Figure S4**- Western Blot for Tubulin corresponding to samples of Figure S3. - Lane 1: control 12-Z, lane 2: MSI-1- and MSI-2-double-knockdown, lane 3 and 4: patient 2 control, lane 5 and 6: patient 2 MSI-1- and MSI-2-double-knockdown*

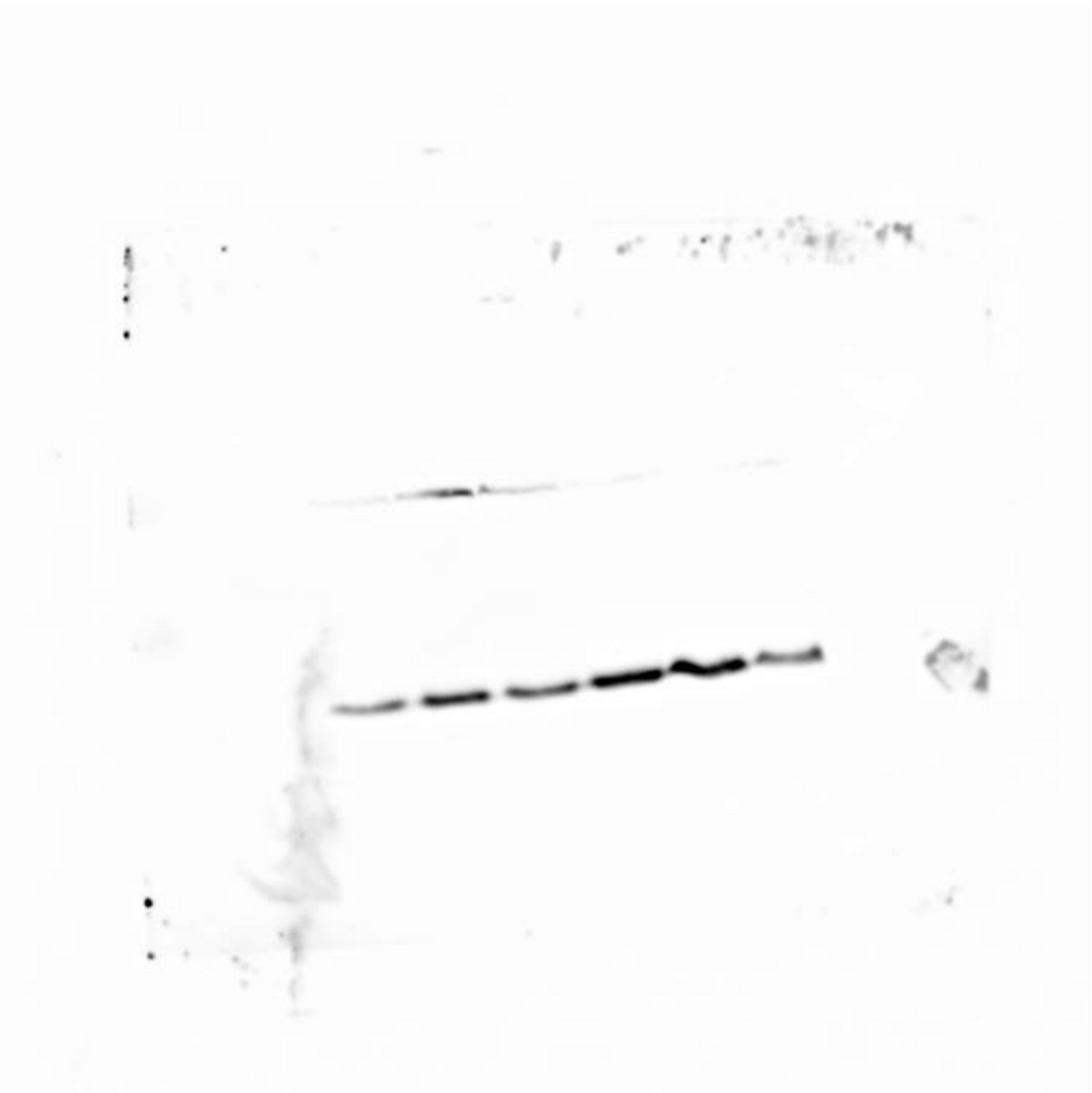

***Supplementary Figure S5-*** *Western Blot for p21 in 12-Z cells - Lane 1-3: control, lane 4-6: MSI-1- and MSI-2-double-knockdown*

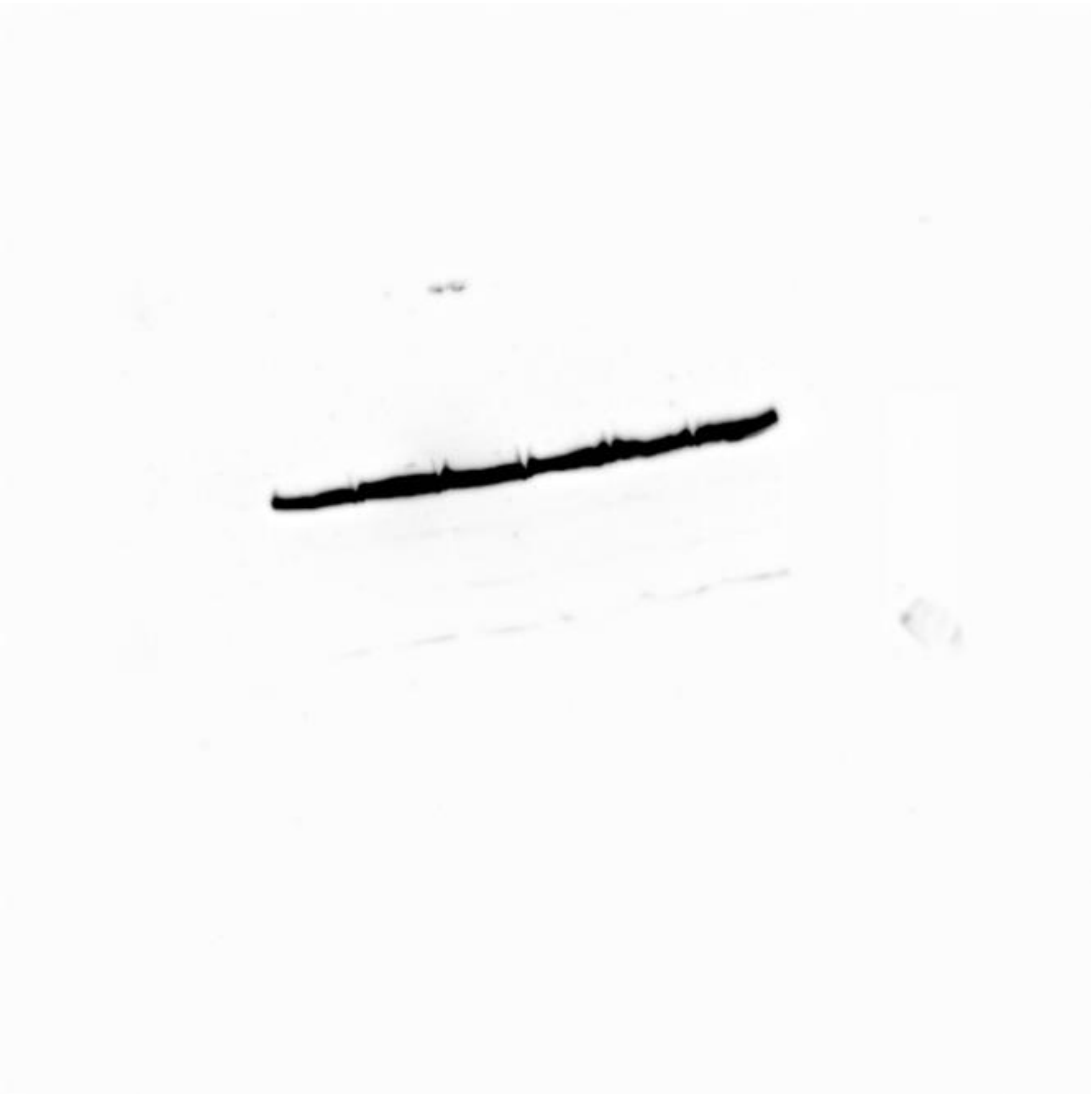

***Supplementary Figure S6**- Western Blot for Tubulin in 12-Z cells, samples correspond to figure S5 - Lane 1-3: control, lane 4-6: MSI-1- and MSI-2-double-knockdown*
